# Medical News and Miscellany

**Published:** 1891-12

**Authors:** 


### Medical News and Miscellany.

**Dr. J. J. Hunter**, of Galveston, was stricken with parrlysis on the 14th of December.

**Married.**—In Austin, Texas, by Rev. T. B. Lee, December 21, Dr. C. J. Smith, the popular Austin dentist, to Mrs. Clara Maillot, of Austin.

**We call attention** of our readers to Messrs, John Wyeth & Brother's new advertisement, which appears in this number, relating to their Compressed Tablets and new remedies (Antipyretics) for Influenza, Neuralgia, Headache, etc., and their great convenience for administration.

**Phillips' Cod Liver Oil Emulsion**, with Wheat Phosphates. Contains 50 per cent. choicest Norway Oil. Is perfectly miscible with water or milk. Has the globules minutely (microscopically) subdivided. Combines the nourishing properties of the

Wheat Phosphates. Is most rapidly absorbed and digested. Is not saponified. Does not nauseate or distress the stomach.

---

It is required by law, in Massachusetts, that in all schools the children shall be instructed in the subject of alcohol and other narcotics; taught their effects upon the economy. This is a wise law, one that should be emulated in every State in the Union. It is the only way we will ever be able to combat the great evil of intemperance. Our correspondent and collaborator, Dr. L. H. Luce, of West Tisbury, Mass., has prepared a book upon the subject, for the use of schools, and has favored us with a copy. It is published by D. C. Heath & Co., Boston.

---

A Doctor Shot Down in the Streets.—Dr. Allen, of Richmond, Texas, formerly of Atlanta, Cass county, Texas, was foully murdered in the streets of Richmond, on the 14th day of December. A rich planter, named Matt Dunlevy, waylaid him, armed with a double-barreled shot gun, and hailing the Doctor as he passed, shot him down, firing both barrels of the gun; not content with that, although Allen was riddled with buck-shot, Dunlevy fired again, this time with a pistol, the ball breaking the neck of the unfortunate young man. The cause for this cruel and cowardly murder is said to have been that Allen was accused of having made disparaging remarks about Dunlevy's daughter.

---

Married. — In San Antonio, Texas, December 9th, Dr. R. H. L. Bibb, of Saltillo, Mexico, to Miss Aline Fischer, of San Antonio.

THE JOURNAL tenders the Doctor its most cordial congratulations, and wishes that all his fondest anticipations may be realized ; that like a tempest-tossed mariner safely moored in a delightful haven, he may find rest and happiness ; and may a happy life of wedded felicity be his. The bride, the JOURNAL would congratulate on a rare conquest. She has captured one of the most accomplished physicians in this day of high professional excellence, and a splendid type of physical manhood and dignity. Amongst the Doctor's vast and ever increasing host of attached friends, none more sincerely rejoice in hearing that he is married, and no one more sincerely wishes him happiuess; he deserves it. But we will never—no, never forgive him for not inviting us to come to San Antonio and witness the happy event.

**Leprosy in the U. S.** — Supervising Surgeon General Wyman, of the Marine Hospital Service, says, in his report to Congress, that there are cases of genuine leprosy in the U. S., and that science being powerless to cure the disease, some steps should be taken to isolate these victims and care for them. He suggests the establishment by the government of a special hospital on some adjacent island belonging to the U. S., and the appointment of medical officers to take charge of it; to this hospital all cases are to be sent. Dr. Wyman calls attention to the fact that no State in the Union has made any provision for the care and isolation of the lepers within their borders.

Four cases of leprosy are reported to have occurred in Texas, one at San Antonio, two at Galveston,—detailed reports of which, by Dr. Dock, were published in the JOURNAL, and one case in Coryell county.

---

**A Valuable Suggestion.**—In this "land of the free and home of the brave," while with a royal hand and loyal heart we extend a wellcome to the oppressed of every nation and every clime, we are asleep to the danger that shadows our land. The annual report of Supervising Surgeon General Walter Wyman, of the marine hospital seıvice, has just been submitted the Secretary of the Treasury, and in it he asks special attention to the prevalence of leprosy in the neighboring West Indies and South America, and also to the presence of the loathsome disease in various cities and localities of the United States, and says cases may be allowed to exist in certain localities because no provision is made for their isolation, and as no State has ever guarded its domain by the erection of a hospital for these incurables, Dr. Wyman suggests that the government take the matter in hand and establish a National leper hospital. It may be that coming ages will reveal some cure for this awful malady, but we are surely recreant to duty in the great matter of alleviating the woes of suffering humanity if we allow such infection to lurk within our borders and make no effort to stay its ravages. We trust our government will act upon the recommendations speedily.

Mention is also made in the report of the successful operation at the eight National quarantine stations at Chandeleur. Fifty vessels infected with yellow fever were held there and disinfected, and not a single case of yellow fever occurred in any of the southern cities; so that such measures are the safeguards of our couutry in avoiding the transmission of disease.—*Statesman.*

### WHAT IS ANTIKAMNIA?

LETTER FROM DR. W. THORNTON PARKER, U. S. A.

The relief of suffering is the object of philanthropy. The relief of pain commands the highest efforts of the physician. Remedies which are useful in the relief of pain are always highly prized, and the discoverer is entitled to the highest honor. For many years numberless remedies have been offered to the profession as analgesics and anodynes; the list is a long one, and contains many products of great reliability; the result of faithful study and experiment. One especially has received the confidence of the profession, the Antipyrin of Knorr; but recently there has appeared a product which bids fair to be a successful rival of this and all others, and in truth to deserve the title, "A Succedaneum for Morphia."

Antikamnia is no longer a stranger to the medical profession, but is daily winning laurels in its mission as "opposed to pain." It is described as a new combination of coal tar derivatives, of the series CnH2n-6 into which the amines have entered, forming the various amido-compounds. It is by the further combination of other organic bodies with the amido-benzoles that many of the valuable antipyretics and analgesics have been brought into existence. Antikamia has as its base the derivatives of the amido-benzoles, so combined as to to obviate the bad effects caused by many of this series of organic bodies when administered alone.

Briefly stated, it is indicated in Cephalalgia, Neuralgia, attacks of Acute Rheumatism, Locomotor Ataxia, Sciatica and the disorders of Menstruation accompanied by pain. In the treatment of Malaria, Typhoid and other fevers, it is fast winning its way. In the treatment of diseases where it is important to exhibit quinine, the action of Antikamia will be found especially desirable in preventing the disturbance of the nervous system so frequent when quinine is given in large quantities.

Several very interesting articles have appeared of late describing its action. Dr. Holland, in the "*Medical Summary*" of May, describes an interesting case of Dysmenorrhœa promptly relieved by its use. My own experience confirms this. I believe it to be one of the best remedies for the relief of pain in this disease. Experience with its use in cases of La Grippe, Asthma, etc., have convinced me of its efficacy. Indeed, to state the merits of Antikamnia more fully it would be necessary to mention all the diseases in which pain is a prominent sympton. It can be used advantageously in the treatment of the various forms of Hysteria, where bromides have been indicated heretofore.

So far as my experience goes, we need not anticipate unfavorable after-effects; its action is soothing, tranquilizing, and diminishes the tendency of a rise of the bodily temperature. Antikamnia has been found by Dr. Alvord, of "The St. Louis City Hospital," eapecially valuable in the treatment of pythisis.

Dr. Gayle, of Kansas City, Mo., reports very satisfactory results from its use in the treatment of Typhoid, in an article published in the "*St. Louis Courier of Medicine*," August, 1890.

A very successful operation, performed by Dr. A. V. L. Brokaw, Demonstrator of Anatomy and Surgery, Missouri Medical College, in a case of a severe stab wound of thorax and abdomen, published in the same journal of December, 1880, shows how valuable is Antikamnia as a remedy for the relief of pain. It is best exhibited in doses of from three to ten grains every three or four hours, in powder or tablet form, taken in water or wine.

Its anodyne action is admirably shown in the treatment of the insomina of neurasthenic patients, and for the treatment of many cases of sleeplessness in over-worked business and professional men.

